# *Sar1b* mutant mice recapitulate gastrointestinal abnormalities associated with chylomicron retention disease

**DOI:** 10.1016/j.jlr.2021.100085

**Published:** 2021-05-05

**Authors:** Nickolas Auclair, Alain T. Sané, Lena Ahmarani, Nathalie Patey, Jean-François Beaulieu, Noel Peretti, Schohraya Spahis, Emile Levy

**Affiliations:** 1 Research Center, CHU Ste-Justine, Université de Montréal, Montreal, Quebec, Canada; 2 Department of Pharmacology & Physiology, Université de Montréal, Montreal, Quebec, Canada; 3 Department of Nutrition, Université de Montréal, Montreal, Quebec, Canada; 4 Department of Pathology, Université de Montréal, Montreal, Quebec, Canada; 5 Laboratory of Intestinal Physiopathology, Department of Immunology and Cell Biology, Faculty of Medicine and Health Sciences, Université de Sherbrooke, Sherbrooke, Quebec, Canada; 6 Department of Pediatric Gastroenterology-Hepatology and Nutrition, Laboratory INSERM 1060 Cardiovascular Metabolism Endocrinology and Nutrition CarMEN, Lyon, France

**Keywords:** S*ar1b*, gene defects, embryonic lethality, chylomicron, dietary fat, lipid metabolism, CRISPR/Cas9, chylomicron retention disease, fatty acid β-oxidation, lipogenesis, ACADL, Acyl-CoA dehydrogenase and long chain, ACC, Acetyl CoA carboxylase, AMPK, AMP-Activated protein kinase, CHOL, Cholesterol, CM, Chylomicron, COPII, Coat protein complex II, CPT1, Carnitine palmitoyl transferase, CRD, Chylomicron retention disease, MTTP, Microsomal triglyceride transfer protein, pACC, Phospho ACC, pAMPK, Phospho AMPK, TG, triglyceride

## Abstract

Chylomicron retention disease (CRD) is an autosomal recessive disorder associated with biallelic *Sar1b* mutations leading to defects in intracellular chylomicron (CM) trafficking and secretion. To date, a direct cause-effect relationship between CRD and *Sar1b* mutation has not been established, but genetically modified animal models provide an opportunity to elucidate unrecognized aspects of these mutations. To examine the physiological role and molecular mechanisms of Sar1b function, we generated mice expressing either a targeted deletion or mutation of human *Sar1b* using the CRISPR-Cas9 system. We found that deletion or mutation of *Sar1b* in mice resulted in late-gestation lethality of homozygous embryos. Moreover, compared with WT mice, heterozygotes carrying a single disrupted *Sar1b* allele displayed lower plasma levels of triglycerides, total cholesterol, and HDL-cholesterol, along with reduced CM secretion following gastric lipid gavage. Similarly, decreased expression of apolipoprotein B and microsomal triglyceride transfer protein was observed in correlation with the accumulation of mucosal lipids. Inefficient fat absorption in heterozygotes was confirmed via an increase in fecal lipid excretion. Furthermore, genetically modified *Sar1b* affected intestinal lipid homeostasis as demonstrated by enhanced fatty acid β-oxidation and diminished lipogenesis through the modulation of transcription factors. This is the first reported mammalian animal model with human *Sar1b* genetic defects, which reproduces some of the characteristic CRD features and provides a direct cause-effect demonstration.

Chylomicron retention disease (CRD) (OMIM # 246700) is an integral part of congenital disorders of intestinal lipid transport (1). It is a rare, autosomal recessive disorder, featured with failure to thrive in infancy, fat malabsorption, chronic diarrhea, loose greasy stools, frequent vomiting, and abdominal swelling. A few patients with CRD sporadically exhibit hepatomegaly with a moderate degree of macrovesicular steatosis and cytolysis (2, 3, 4, 5). Ataxia, proprioceptive abnormalities, sensory neuropathy, decreased bone density, cardiomyopathy, muscular pain, and ophthalmological manifestations are additional complications observed in some subjects with CRD.

Biallelic mutations in the *SARA2* or *SAR1B* gene characterize patients with CRD (6, 7). The gene encodes a guanosine triphosphatase (*SAR1B*), an essential factor of the coat protein complex II (COPII), ensuring vesicular traffic from the endoplasmic reticulum (ER) to the Golgi apparatus. As reported by elegant studies, the Sar1b enzyme recruits the COPII complex heterodimers (i.e., Sec23/24 and Sec13/31) to the ER membrane, to form pre-chylomicron (CM) transport vesicles (8, 9, 10, 11). The latter efficiently transpose their pre-CM cargo to the Golgi, before being targeted to the basolateral site of the enterocyte in order to enter blood via the lymphatic duct. Therefore, genetic *SAR1B* defects are expected to result in CM secretion failure.

Approximately 60 patients with CRD have so far been described with frameshift, splice site, and missense mutations affecting the *SAR1B* gene, located on chromosome 5q31.1. The pioneering exploration of genetic defects in eight families disclosed three frameshift (75-76 delTG, 555-558 dupTTAC, 349-1G>C) and five missense mutations (109G>A, 409G>A, 537T>A, 536G>T, 542T>C) (6). The second exploration of the genetic *SAR1B* abnormalities from eight families identified three new molecular aberrations: a stop codon mutation (364G>T), a 5,946 bp deletion (total exon 2), and a missense mutation (554G>T) (12). Subsequently, a stop codon mutation (G19T) was brought out in exon 7 (13) while two additional mutations were detected in exon 2 (G11D) and exon 4 (D75G) (14). More recently, a novel homozygous deletion at position 142 (c.142delG) gave rise to a frameshift-derived premature stop codon, 17 amino acids further on (p.Asp48ThrfsX17), thereby resulting in a truncated protein (15). Further studies on Tunisian children with CRD reported a novel nucleotide transition in *Sar1b* exon 4 (c.184G>A), resulting in a nonconservative amino acid substitution (p.Glu62Lys) (16). D137N was the most prevalent *SAR1B* gene mutation found in French Canadians, which resides in the highly conserved guanine-nucleotide binding motifs (G′xG^37^KT^39^ and NKxD^137^) of the small GTPases (17). The D137N mutation breaks the guanine H-bond, thereby abolishing the guanine recognition site and damaging the protein (18). Apparently, truncated and nonfunctional proteins are produced by nonsense mutations and the whole deletion of exon 2, which are predicted to modify the affinity of Sar1b protein for GDP and GTP, thereby affecting its interaction with the endoplasmic reticulum and COPII machinery components.

Although CM secretion was significantly decreased in patients with CRD, recent studies in genetically modified *Sar1b* in intestinal Caco-2/15 cells emphasized the need to double knockout (KO) the *Sar1b* and its *Sar1a* paralog in order to abolish CM secretion (19). Whether the discrepancy is due to the colonic tissue and cancer origin of the parental Caco-2 cells is completely unknown. In McArdle-RH7777 hepatocytes, Sar1b expression increased apolipoprotein (Apo) B-containing lipoproteins, while his Sar1a paralog antagonized Sar1b's Apo B-lipoprotein secretion (20). Of interest, in zebrafish, the tissue distribution of *Sar1b* and *Sar1a* was different during development while *Sar1b*-deficient embryos accumulate dietary lipids in enterocytes and displayed abnormal formation of craniofacial cartilage and absence of neuroD-positive neurons in embryo brain (21). Quite recently, Sar1b has been reported to be crucial to radial migration and axon morphogenesis of the cortical neurons (22).

Although the above studies contributed to the advancement of our scientific knowledge about the Sar1b physiological functions, additional proof-of-principle experiments are highly needed to scrutinize the specific role of Sar1b. In particular, greater effort is needed to understand the mechanisms of Sar1b not only on CM assembly and delivery failure, but also on typical CRD hypocholesterolemia and HDL-C decrease (2, 12, 23, 24). Moreover, whether human *SAR1B* mutations and deletions differently affect intracellular lipid homeostasis (e.g., fatty acid [FA] β-oxidation and lipogenesis) remain to be explored. Finally, additional puzzling issues remain unanswered, and among them: Is it possible to engineer mice with full Sar1b deficiency, even though past studies observed important abnormal brain development in congenital malabsorption syndromes, or does the KO lead to lethal embryos? Are half-normal levels of the Sar1b mRNA and protein insufficient to maintain normal phenotype? Does the intestine and liver differ in their lipid metabolism in response to defects in Sar1b? To gain insight into most of the above issues, we attempted to generate a CRD mouse model using the CRISPR-Cas9 editing system by separately introducing a deletion and a point mutation identified in patients.

## Materials and Methods

### Generation of Sar1b KO (*Sar1b*^*del/+*^) and mutated (*Sar1b*^*mut/+*^) mice

Heterozygous male and female *Sar1b*^*del/+*^ and *Sar1b*^*mut/+*^ mice were obtained with the CRISPR-Cas9 technology at the Centre Hospitalier de l’Université de Montréal (CRCHUM), as broadly described in the supplementary file. In particular, CRISPR-Cas9 editing technology was used to *i*) generate a deletion of 545 base pairs removing *Sar1b* exon 2 on chromosome 11 (GRCm38/mm10, chr11:51, 777, 181-51, 777, 725 Del) (*Sar1b*^*del/+*^), or *ii*) substitute G to A on chromosome 11 (GRCm38/mm10, chr11: 51, 789, 257G>A). This mutation results in the substitution of an aspartic acid (D) to an asparagine (N) amino acid at position 137 (D137N) (*Sar1b*^*mut/+*^). To this end, two synonymous mutations were introduced in the mouse generation process: *1*) a C to T substitution at position 51 (GRCm38/mm10, chr11: 51, 789, 256C>T) was incorporated to facilitate the genotyping procedure (introduction of a *Msel* restriction site), and *2*) a C to G substitution at position 51 (GRCm38/mm10, chr11: 51, 789, 271C>G) was introduced to mutate the PAM site and avoid repair template cleavage by the cas9 endonuclease. The heterozygous mice with similar mutation or deletion were intercrossed to produce the homozygous mice. Genetically modified mice were housed individually and fed ad libitum with a standard chow diet. Body weight and food intake were measured every day. All experimental procedures performed in the study were approved by the Institutional Animal Care Committee of Ste-Justine Hospital.

### Determination of postprandial chylomicron secretion

Following a 6-h fast, blood was taken from the tail vein. Immediately after this procedure, male and female mice received a 200 μl oral gavage of olive oil or 4 μCi [^14^C]-triolein. Ten minutes later, animals were injected with an intraperitoneal dose (1 mg/kg) of Pluronic F-127 (10% in saline) to inhibit lipoprotein lipase activity and prevent CM catabolism. Blood (50 μl) was transferred into EDTA-containing tubes at times 60, 90, and 120 min after olive oil gavage. Mice were sacrificed, and blood was collected. Plasma and isolated tissues were flash-frozen and kept at −80°C for further analyses.

### Chylomicron isolation

CMs were separated by ultracentrifugation using an MLA-130 rotor as described previously (25). The upper fraction was collected, and its lipid moieties, triglycerides (TGs), and cholesterol (CHOL), were determined by commercial kits (Wako Diagnostics, USA). Scintillation liquid was added to 200 μl of each fraction moiety to determine the radioactivity derived from the labeled [^14^C]-triolein, using a β counter.

### Biochemical analysis

As above, commercial kits were used to analyze insulin (Mercodia), TG, and CHOL in plasma. HDL-C was analyzed by precipitation with polyethylene glycol 6000 as previously described with some modifications (26). Briefly, plasma (10 μl) was mixed with polyethylene glycol at a ratio 1:1 and allowed to rest at room temperature for 20 min. Then after, samples were centrifuged at 4,600 rpm for 40 min, the upper phase was harvested, and CHOL was measured.

### Lipid analysis

Approximately 0.1 g of proximal intestine and liver tissues were homogenized in 1 ml of EDTA buffer, and lipids were extracted in a 2:1 chloroform/methanol solution overnight at 4°C. After evaporation of the lower phase, lipids were resuspended in 400 μl of H_2_O, and TG and CHOL were determined. For fecal lipids, feces were collected on isopad over a 24-h period. Feces (50 mg) were dried at 70°C and extracted with chloroform/methanol before lipid determination as described above.

### Liver and intestinal histology

Tissues were fixed in formalin (10%) and embedded in paraffin. Thereafter, they were processed for light microscopy with hematoxylin-eosin. Images of stained tissues were captured by a Zeiss Imager A1. Measurements were taken with the axiovision software.

### Western blot analysis

Intestinal and liver tissues were prepared for Western blotting as described previously (25).

Proteins were separated on 8% SDS-PAGE gel and electroblotted onto nitrocellulose membranes. Nonspecific binding sites of the membranes were blocked using defatted milk proteins, followed by the addition of primary antibodies directed against acetyl CoA carboxylase (ACC), carnitine palmitoyl transferase a (CPT1a), fatty acid synthase, AMP-activated protein kinase α (AMPKα) and phoshpoAMPK^Thr172^ (pAMPKα) (1/1,000, Cell signaling), GAPDH, ABCA1, peroxisome proliferator activated receptor coactivator 1α (PGC-1α) (1/1,000, Abcam), Apo A1 (1/5,000, Abcam), microsomal triglyceride transport protein (MTTP), acyl-CoA dehydrogenase long chain (ACADL) (1/1,000, Thermo Fisher Scientific), PPAR-α (1/1,000, Cayman Chemical), phospho acetyl CoA carboxylase^Ser79^ (pACC) (1/1,000, Millipore), Apo B (1/1,000, Sigma-Aldrich), and β-actin (1/250,000, Sigma-Aldrich). Bands were captured with the Chemidoc Imaging System and analyzed with the ImageLab software (Bio-Rad). For every protein of interest, β-actin or GADPH was used for sample normalization.

### RNA extraction and RT-qPCR analysis

RNAs from flash-frozen jejunum and liver were extracted using Trizol reagent according to the manufacturer’s instructions (Ambion). RNA concentration and purity were measured using a Biodrop spectrophotometer (Montreal Biotech Inc.), and the ratio absorbance at 260 and 280 nm was used to determine purity as described previously (19, 27). The thermal profile included an initial denaturation step at 95°C for 30 s, followed by 40 cycles of denaturation at 95°C for 3 s and annealing and extension at 60°C for 30 s. Amplified genes were quantified by fluorescence using the PowerUp SYBR Green Master Mix (Life Technologies). Levels of expression of target-gene mRNAs were calculated by the 2^−ΔΔCT^ method (19). The list of all primers used is found in the supplementary file.

### Statistical analysis

Results are presented as mean ± SEM. Since each group of genetically modified mice was compared with the WT group, data were evaluated by an unpaired sample two-parametric *t-*test. GraphPad Prism 8.0 software (GraphPad Software, CA) was used for all statistical analysis. Significance is considered at *P* < 0.05.

## Results

### Generation of *Sar1b*^*del/+*^ and *Sar1b*^*mut/+*^ mice

**Fig. 1:**
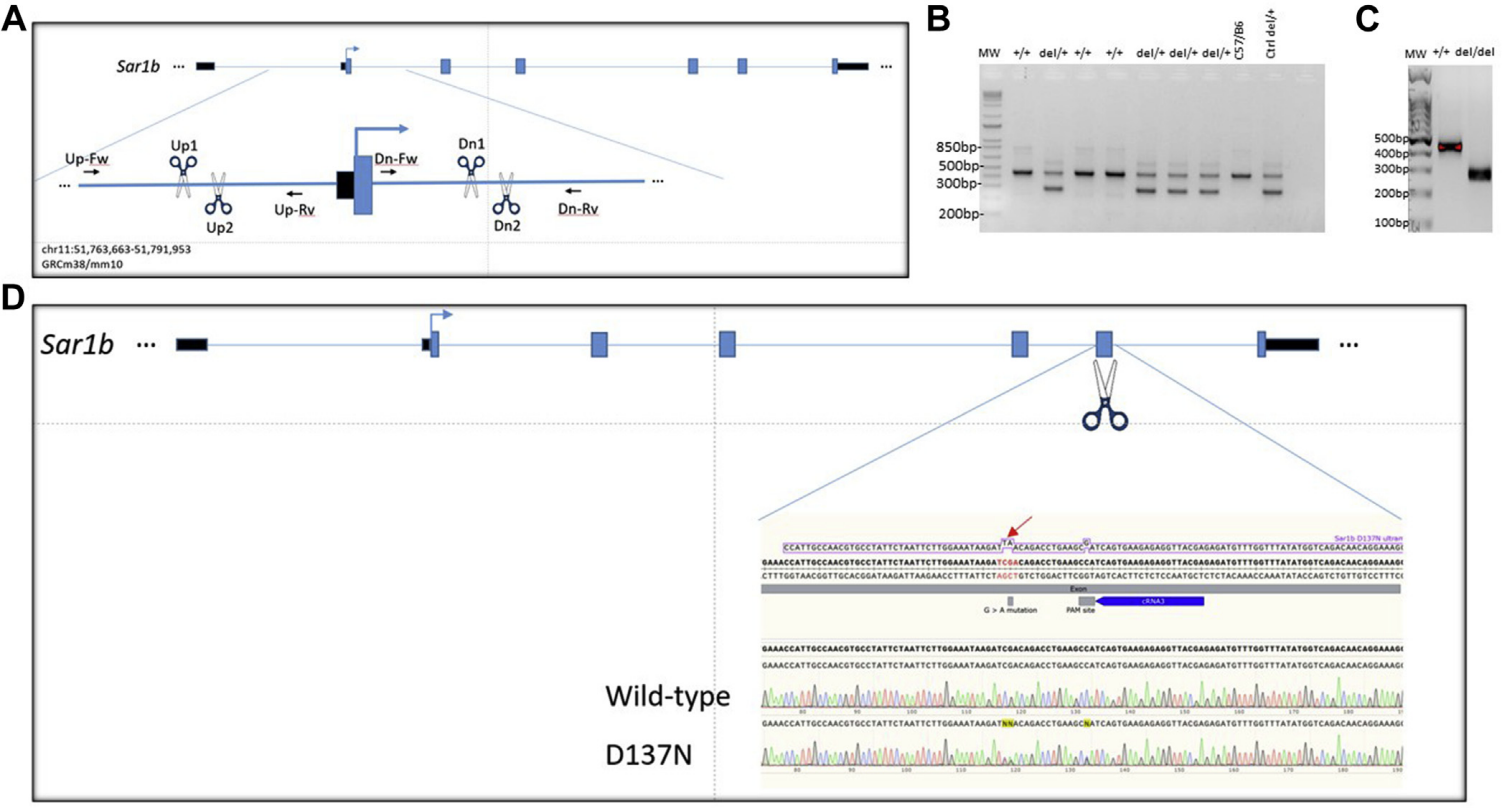
Targeted disruption and point mutation of *Sar1b* gene. A: Map of the *Sar1b* locus together with the targeted disruption of *Sar1b* strategy. Exons (blue filled boxes), introns (thin line), and start of the Sar1B ORF (arrow) are indicated. CRISPR-Cas9 was successfully used to ablate *Sar1b* exon 2 using crRNA_Up1 ACAACAAGTCCCTGTTACCCAGG, crRNA_Up2 TAGATGAGGTTCTATCAGCC, crRNA_Dn1 AAATCACGTAATCGTAGGCCAGG, and crRNA_Dn2 TACGATTACGTGATTTCAAGAGG. B and C: Genotyping for deletion and zygosity. Analysis of gene-targeting events was performed by PCR assay of purified genomic DNA demonstrating wild-type, heterozygous, and homozygous mice for the *Sar1b* KO mutation. Wild-type (+/+) mice typically exhibit an intense 439 bp fragment, whereas a supernumerary 275 bp fragment, only present in homozygous (−/−), is distinguishable in heterozygous (+/−) mice. D: Map of the *Sar1b* locus together with the targeted point mutation of *Sar1b* strategy. Exons (blue filled boxes), introns (thin line), and start of the *Sar1b* ORF (arrow) are indicated. CRISPR-Cas9 was successfully used to introduce a d.409G. to a nAsp137Asn (arrow) mutation in *Sar1b*. The 405 bp sequence encompassing the point mutation was amplified by PCR with forward primer GACTGAGTCCTTGGCTATTTGG and reverse primer CCTTTCTTAAGCTGGGTATGGA. The chromatograms compare the original sequence from gene bank (NM_025535.2) with the ones of a wild-type mouse and a D137N point mutation. Introducing the mutation (CG to TA) introduced a MseI restriction site without affecting the isoleucine amino acid and has mutated the aspartic acid amino acid to an asparagine in position 137.

To generate mice with deleted or mutated *Sar1b*, a specific region of *Sar1b* was targeted as shown in Fig. 1A, D, respectively. Genotyping of postnatal offspring from both altered *Sar1b* heterozygous intercrosses revealed the total absence of viable mouse homozygous for the *Sar1b* for both alterations, indicating embryonic lethality during gestation (Table 1). In order to exclude possible susceptibility due to genetic C57BL/6 background, we examined whether the 129/sv mouse strain was more amenable to produce live *Sar1b*^*-/-*^, since, in some circumstances, this strain has been found to be more resistant to several diseases (28, 29, 30). There again, no live and dead homozygotes were observed.

**Table 1:** Results of heterozygous mice breeding

		Sar1b genotype		
Genotypes of offspring from intercross of heterozygous *Sar1b*^*del/+*^ mice				
Mating	Total pups	*+/+*	*del/+*	*del/del*
Intercross (♂C57BL/6^*del/+*^ × ♀C57BL/6^*del/+*^)	57	18 (32%)	32 (56%)	7^a^ (12%)
Intercross (♂sv129^*del/+*^ × ♀sv129^*del/+*^)	11	7 (64%)	4 (36%)	0

Genotypes of offspring from intercross of heterozygous *Sar1b*^*mut/+*^ mice				
Mating	Total pups	*+/+*	*mut/+*	*mut/mut*
Intercross (♂C57BL/6^*mut/+*^ × ♀C57BL/6^*mut/+*^)	69	13 (19%)	56 (81%)	0
Intercross (♂sv129^*mut/+*^ × ♀sv129^*mut/+*^)	14	7 (50%)	7 (50%)	0

The predicted proportions of *Sar1b* genotypes (*del/+; del/del*) and (*mut*/*+*; *mut*/*mut*) for intercrosses are 25:50:25, but they are distorted in this case because of embryonic lethality and cannibalism of dead pups.

a Pups were dead at birth.

**Fig. 2:**
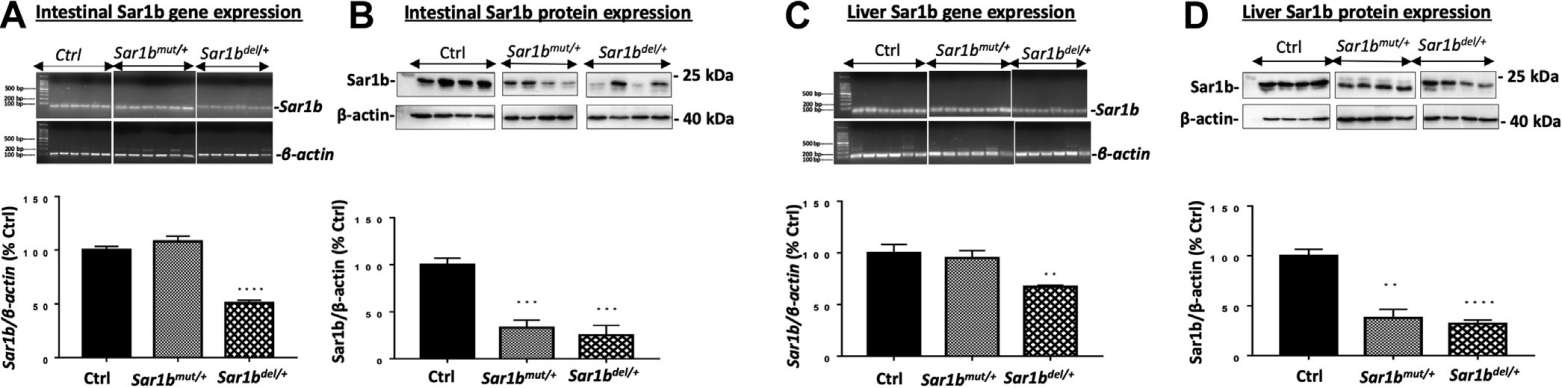
Protein expression of Sar1b in the small intestine and liver of *Sar1b*^*mut/+*^ and *Sar1b*^*del/+*^ mice. Wild-type, *Sar1b*^*mut/+*^, and *Sar1b*^*del/+*^ female and male mice (9–11 weeks) were fed ad libitum with a conventional chow diet for 1 week. Prior to the sacrifice, mice were fasted 6 h, and their tissues were flash frozen. Sar1b (A and C) gene and (B and D) protein expressions were assessed by RT-qPCR and Western blot, respectively, in the intestine and liver. Results represent the means ± SEM of 4–6 mice in each group. ∗∗*P* < 0.01, ∗∗∗*P* < 0.001, ∗∗∗∗*P* < 0.0001 versus Wild-type mice (Ctrl).

Fig. 1B, C show the typical electrophoretic band profile after genotyping of offspring from *Sar1b*^del/+^. To validate the hypothesis that *Sar1b* deletion and mutation produce a lethal phenotype in homozygous mice, the cages of pregnant animals were inspected daily, and any dead pups were removed. The dead pups were genotyped, and they were all found homozygous for the deletion, indicating late-gestation lethality and the inability of producing homozygous viable mice. As to the point mutation model, there was no living or dead pup with the mutation on both alleles, suggesting early embryonic lethality. On the other hand, screening the intestine and liver of living adult mice revealed half level of Sar1b gene and protein expression in heterozygous mice compared with WT mice (Fig. 2A–D).

### Anthropometric and biochemical analyses of *Sar1b*^del/+^ and *Sar1b*^mut/+^ mice

**Fig. 3:**
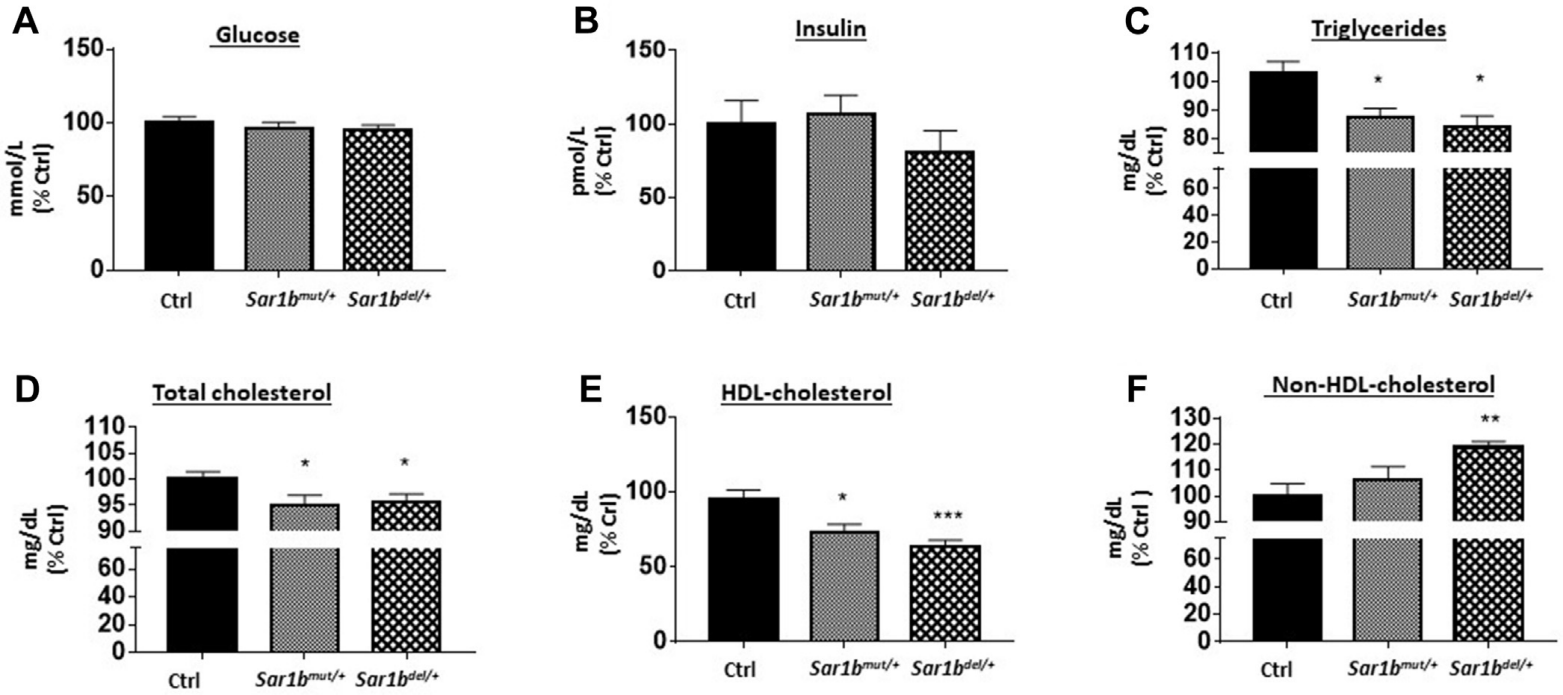
Consequences of *Sar1b* mutation and deletion on biochemical parameters. Wild-type, *Sar1b*^*mut/+*^, and *Sar1b*^*del/+*^ female and male mice (9–11 weeks) were fed ad libitum with a conventional chow diet for 1 week. Before the sacrifice, mice were fasted for 6 h, followed by the recovery of 50 μl of blood from the tail vein. Fasting plasma (A) glucose, (B) insulin, (C) triglycerides, (D) total cholesterol, and (E) HDL-cholesterol concentrations were determined as described in the Materials and Method section. F: Non-HDL-cholesterol was also calculated. Results represent the means ± SEM of 10–13 mice in each group. ∗*P* < 0.05, ∗∗*P* < 0.01, ∗∗∗*P* < 0.001 versus wild-type mice (Ctrl).

No marked changes were noted in energy intake, body weight, and weight of various tissues (supplemental Fig. S1) as well as the plasma glucose and insulin concentrations (Fig. 3A, B) in genetically modified male and female *Sar1b* mice. However, plasma lipid profile analysis showed a significant drop of TG, CHOL, and HDL-C (Fig. 3C–E) levels with an increase in non-HDL-C (Fig. 3F).

### In vivo intestinal fat absorption as a function of *Sar1b* heterozygosity

**Fig. 4:**
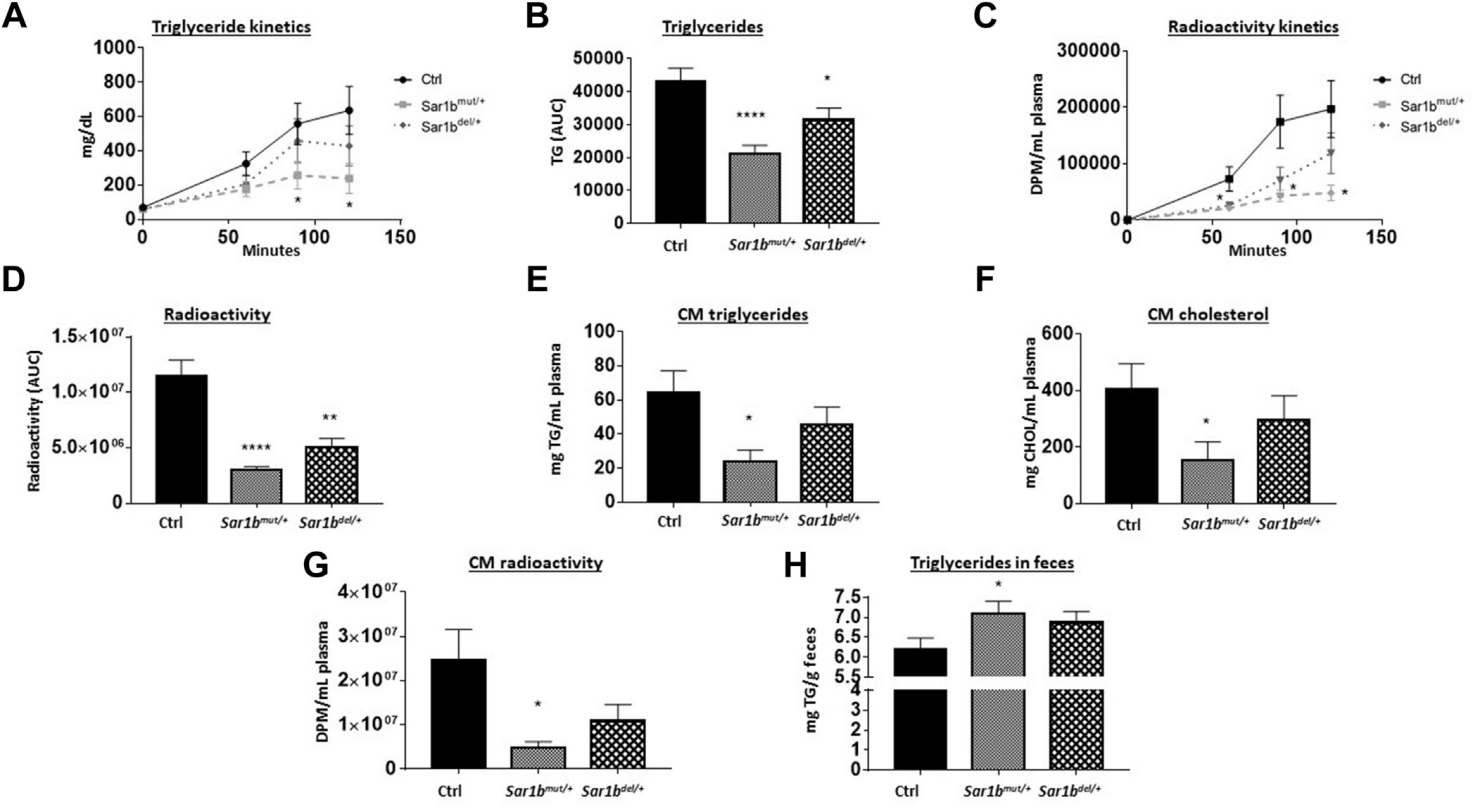
Intestinal fat absorption in *Sar1b*^*mut/+*^ and *Sar1b*^*del/+*^ mice. Wild-type, *Sar1b*^*mut/+*^, and *Sar1b*^*del/+*^ female and male mice (9–11 weeks) were fed ad libitum with a conventional chow diet for 1 week. Prior to the sacrifice, mice were fasted 6 h and then were given 200 μl of olive oil and 4 μCi [^14^C]-triolein by oral gavage. Ten minutes later, mice were peritoneally injected with 1 mg/kg of Pluronic F-127 to inhibit lipoprotein lipase-induced chylomicron catabolism. Blood was taken at 60, 90, and 120 min time points. Plasma (A, B) triglycerides and (C, D) radioactivity derived from the labeled [^14^C]-triolein were determined as described in the Materials and Method section. At 120 min, chylomicrons (CM) content was determined by ultracentrifugation. E: Triglycerides, (F) total cholesterol, and (G) CM scintillation counts were analyzed in this fraction. Finally, feces were dried and (H) triglycerides were measured as described previously. Results represent the means ± SEM of 10–13 mice in each group. ∗*P* < 0.05, ∗∗*P* < 0.01, ∗∗∗∗*P* < 0.0001 versus wild-type mice (Ctrl).

To examine the effects of genetic *Sar1b* modifications on lipid absorption, plasma TG concentrations were measured at different time points after the olive oil intragastric administration (Fig. 4A, B). Compared with controls, *Sar1b*^mut/+^ and *Sar1b*^del/+^ mice had a reduced plasma TG concentration, and the severity of fat malabsorption was more striking in *Sar1b*^mut/+^ mice (Fig. 4C). A similar trend was noticed with [^14^C]-triolein gavage, thereby confirming defective intestinal TG transport (Fig. 4D).

Furthermore, the quantification of lipid content in CM revealed a lower TG (Fig. 4E) and total CHOL (Fig. 4F) content, indicating depressed CM output in genetically modified *Sar1b* mice. A similar modulation was observed with [^14^C]-triolein treatment (Fig. 4G), suggesting the inability of *Sar1b*^*mut/+*^ and *Sar1b*^*del/+*^ mice to export alimentary lipids in the form of CM. Finally, we examined the intestinal fat malabsorption by the estimation of lipids following stool collection. A significant increase in fecal TG was detected in *Sar1b*^*mut/+*^ compared with control mice (Fig. 4H). Nevertheless, only a marginal fat augmentation was noted in *Sar1b*^*del/+*^ mice. Therefore, there is a consistent evidence that *Sar1b* genetic modification results in defects in intestinal lipid transport due to inefficient CM formation, particularly in *Sar1b*^mut/+^ mice.

### Genetic alteration of *Sar1b* in mice impairs key players of intestinal CM assembly and HDL biosynthesis

**Fig. 5:**
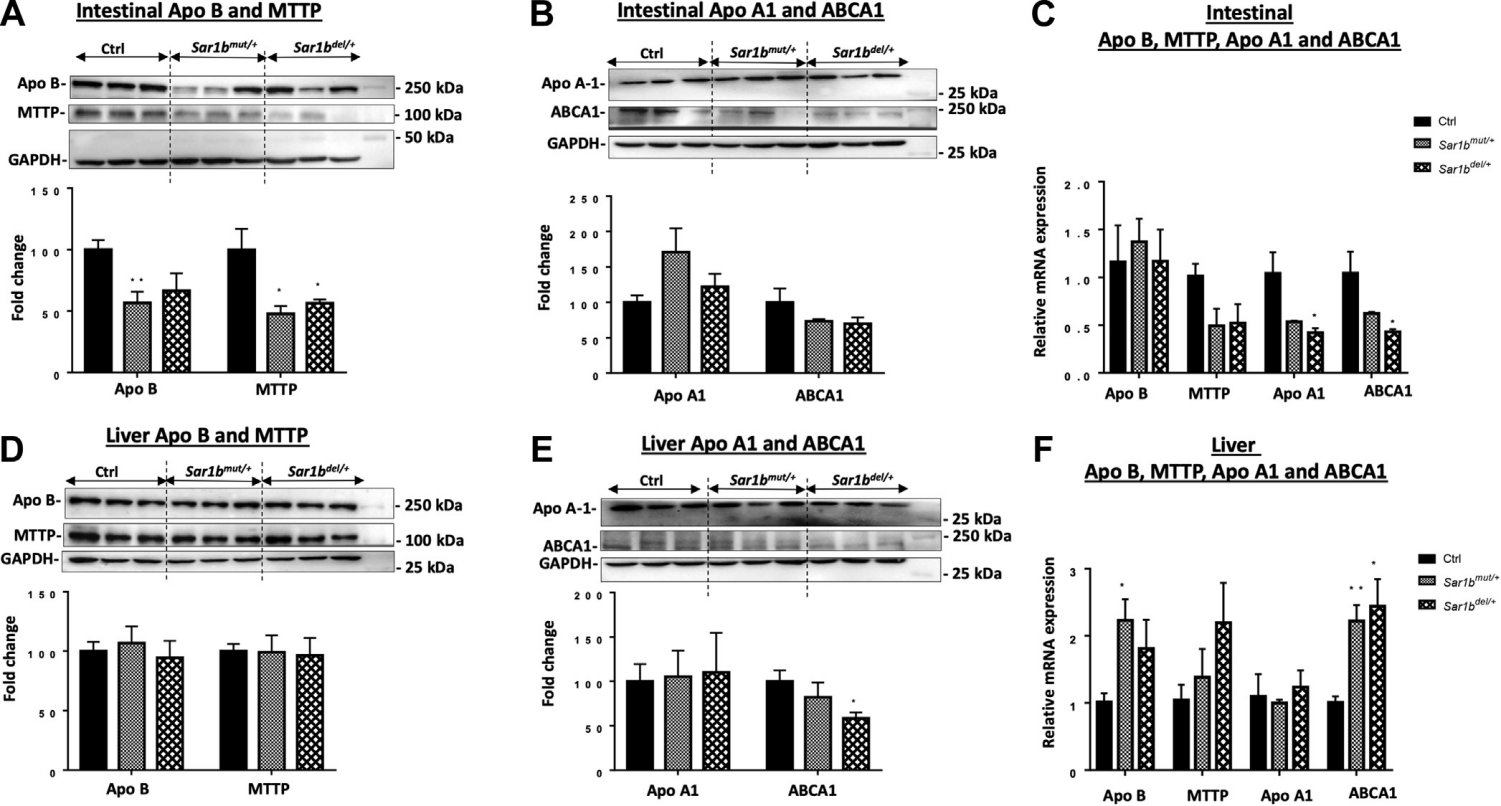
*Sar1b* genetic defects impair key players of intestinal chylomicron assembly and HDL biosynthesis. Wild-type, *Sar1b*^*mut/+*^, and *Sar1b*^*del/+*^ female and male mice (9–11 weeks) were fed ad libitum with a conventional chow diet for 1 week. Prior to the sacrifice, mice were fasted 6 h and then were given 200 μl of olive oil and 4 μCi [^14^C]-triolein by oral gavage. Intestinal and liver tissues were collected. Protein expressions of (A, D) Apo B, MTTP, (B, E) Apo A1, and ABCA1 were analyzed by Western blot in the intestine and liver tissues, respectively. The gene expression was also assessed in (C) the intestine and (F) liver by RT-qPCR as described in the Materials and Method section. Results represent the means ± SEM of 3–6 mice in each group. ∗*P* < 0.05, ∗∗*P* < 0.01 versus wild-type mice (Ctrl).

The aforementioned findings pointed out perturbations in CM output as a result of *Sar1b* disruption. The next logical step was to determine whether *Sar1b* defects had an impact on critical proteins for CM assembly (Apo B and MTTP) as well as HDL biosynthesis (Apo A-I and ABCA1). We therefore analyzed their protein and gene expressions in intestinal and liver tissues. Figure 5 documents a significant reduction of Apo B and MTTP (Fig. 5A) with no apparent differences in Apo A-I and ABCA1 (Fig. 5B) protein expression in the small intestine of *Sar1b*^*mut/+*^ and *Sar1b*^*del/+*^ mice. In contrast, the gene expression displayed a significant drop of Apo A-I and ABCA1 without changes in Apo B and MTTP mRNA levels (Fig. 5C). Moreover, analyzes of liver tissues of *Sar1b*^*mut/+*^ and *Sar1b*^*del/+*^ mice showed a significant decrease in ABCA1 protein mass (Fig. 5E) and an increase in Apo B and ABCA1 mRNA expression (Fig. 5F) without modulation of other key proteins.

### Intestinal and liver lipid content following fat load

**Fig. 6:**
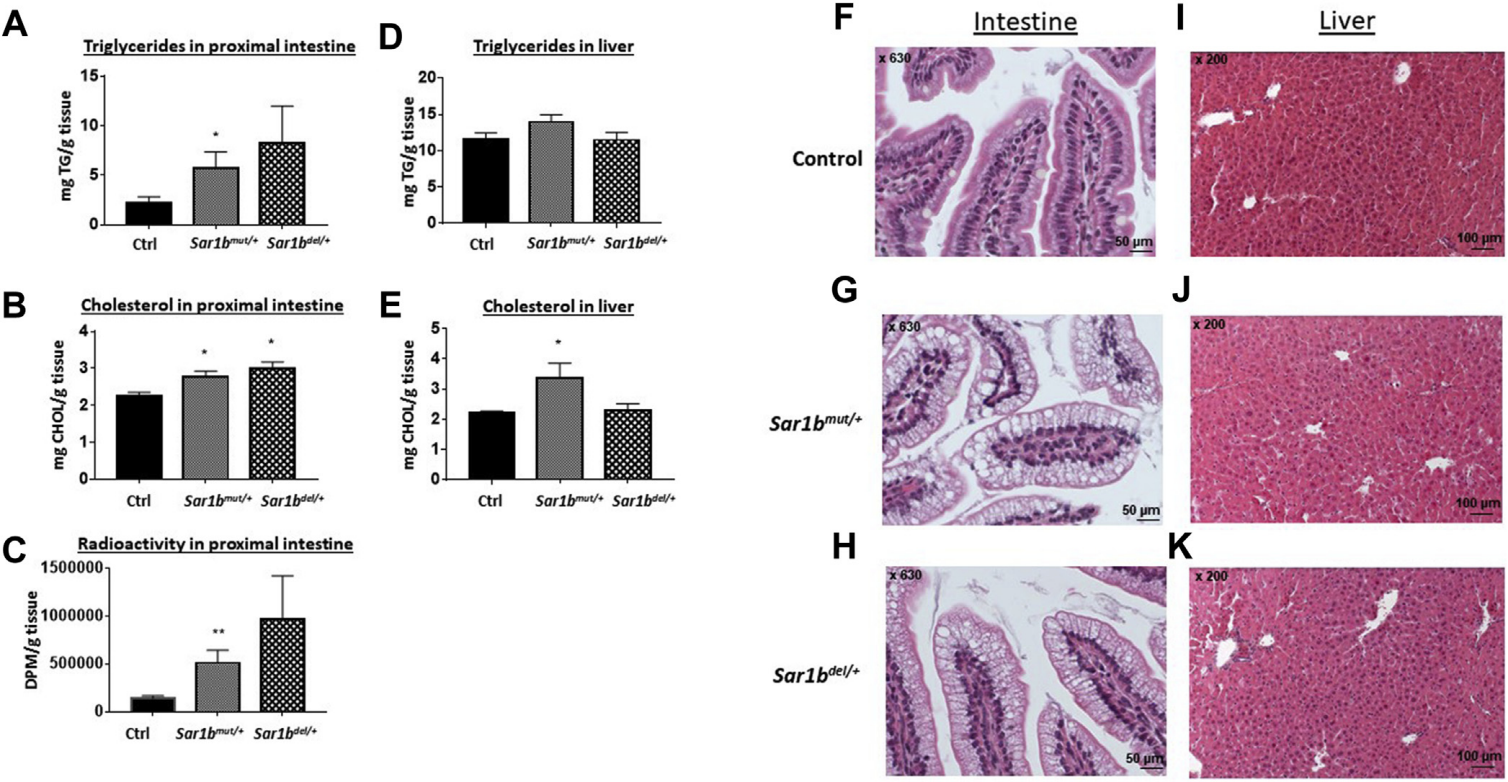
Effect of *Sar1b* genetic defects on intestinal and hepatic lipid accumulation. Wild-type, *Sar1b*^*mut/+*^, and *Sar1b*^*del/+*^ female and male mice (9–11 weeks) were fed ad libitum with a conventional chow diet for 1 week. Prior to the sacrifice, mice were fasted 6 h and then were given 200 μl of olive oil and 4 μCi [^14^C]-triolein by oral gavage. A and D: Triglycerides and (B, E) total cholesterol were extracted from the gut and liver, and the same for (C) radioactivity derived from the labeled [C^14^]-triolein. They were all analyzed as described in the Materials and Method section. Finally, monographs of histology with hematoxylin-eosin were analyzed and expressed in the (F, G, H) intestine and (I, J, K) liver. Results represent the means ± SEM of 10–13 mice in each group. ∗*P* < 0.05, ∗∗*P* < 0.01 versus wild-type mice (Ctrl).

Since genetic *Sar1b* modifications affected intestinal fat absorption through abnormal CM output, lipid retention was assessed in the small intestine and liver. An increase was observed in TG (Fig. 6A), total CHOL (Fig. 6B), and radioactivity (Fig. 6C) in the gut of *Sar1b*^mut/+^ and *Sar1b*^del/+^ mice with a more marked difference in *Sar1b*^mut/+^ mice. As for the liver, only *Sar1b*^*mut/+*^ mice showed a total CHOL accretion (Fig. 6D, E). The biochemical TG results were corroborated by histopathological hematoxylin-eosin staining in the intestine (Fig. 6F–H) and the liver (Fig. 6I–K).

### *Sar1b* heterozygosity altered intestinal but not liver lipid metabolism

**Fig. 7:**
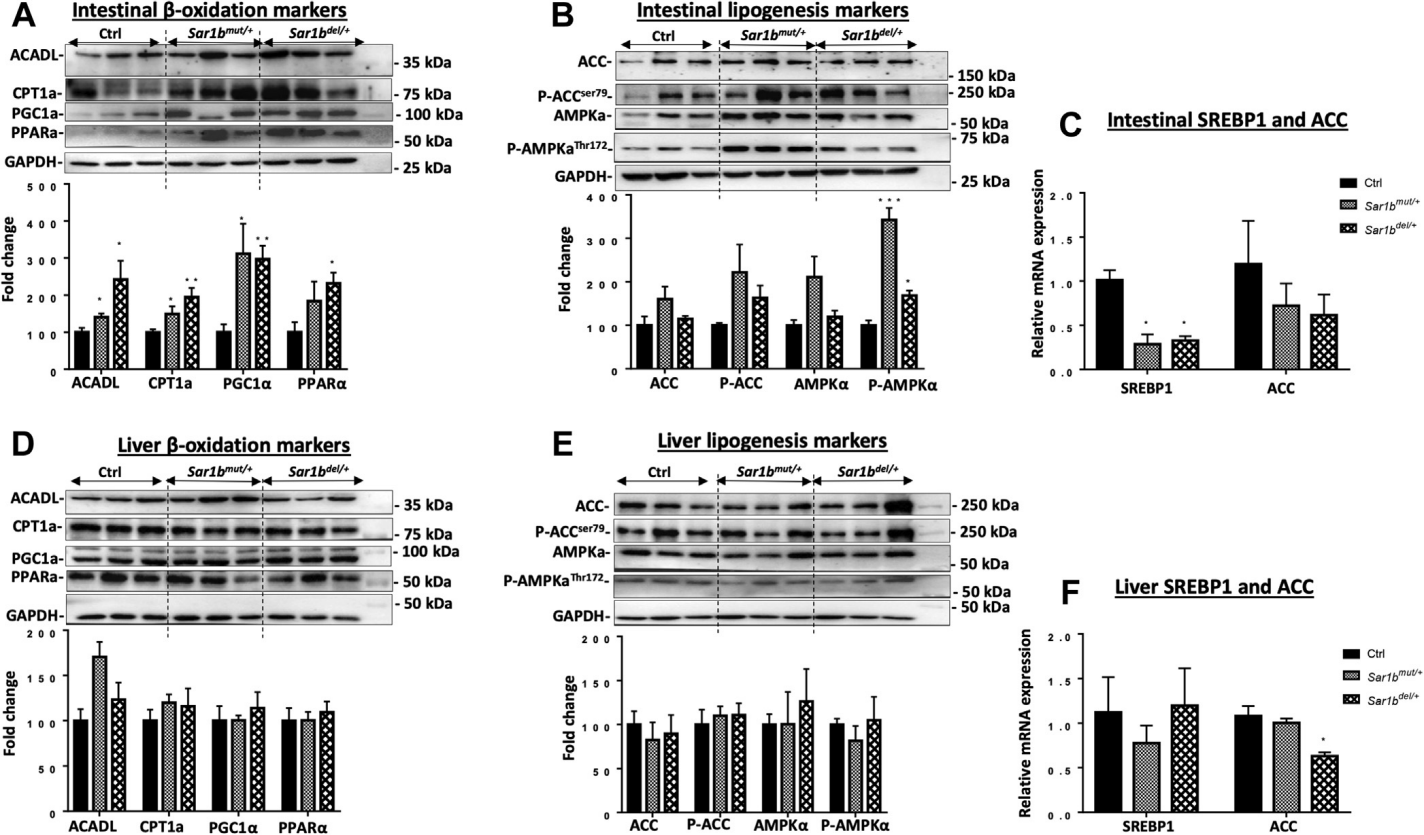
Impact of *Sar1b* genetic defects on intestinal and liver lipid metabolism. Wild-type, *Sar1b*^*mut/+*^, and *Sar1b*^*del/+*^ female and male mice (9–11 weeks) were fed ad libitum with a conventional chow diet for 1 week. Prior to the sacrifice, mice were fasted 6 h and then were given 200 μl of olive oil and 4 μCi [^14^C]-triolein by oral gavage. Protein expression of important markers of (A, B) fatty acid β-oxidation (ACADL, CPT1a, PGC1α, and PPARα) and (D, E) lipogenesis (ACC, P-ACC, AMPKα, and P-AMPKα) were analyzed in the intestine and liver by Western blot as described in the Materials and Method section. The gene expression was also assessed in (C) intestine and (F) liver by RT-qPCR as described in Materials and Method section. Results represent the means ± SEM of 3–6 mice in each group. ∗*P* < 0.05, ∗∗*P* < 0.01, ∗∗∗*P* < 0.001 versus wild-type mice (Ctrl).

CRD is highly characterized by impaired intestinal fat transport owing to the limitation of CM assembly and secretion, and we hypothesized that *Sar1b* deficiency may lead to disturbed lipid homeostasis in the gastrointestinal tract. To this end, the protein and gene expressions of key factors in lipid metabolism were analyzed. Our data revealed a plausible enhancement of FA β-oxidation in the intestine via the upregulation of the mitochondrial enzymes ACADL and CPT1a (Fig. 7A) as well as the protein mass of the powerful transcription factors PPAR-α and PGC-1α, responsible for the modulation of FA homeostasis. (Fig. 7A). On the other hand, AMPKα, pAMPKα, and pACC (Fig. 7B) were conversely modulated in the small intestine of *Sar1b*^mut/+^ and *Sar1b*^del/+^ mice. This was supported by the diminution of SREBP-1 gene expression in the intestine of genetically modified mice (Fig. 7C), pointing out a decline in the lipogenesis process. With respect to the liver, no drastic alterations were noticed either in the protein or mRNA levels of both genetic mouse models (Fig. 7D–F).

## Discussion

As remarkable progress in genetic manipulation allows the generation of powerful animal models for understanding the pathogenesis and modelling of human disease, we followed the same experimental strategy to probe and decipher various aspects of CRD. Using the genome-editing CRISPR-Cas9 approach, we generated two mutant mice, one with the deletion and the other with a point mutation of the *Sar1b* gene. Our first finding was that genetic modification of mouse *Sar1b* results in embryonic lethality of homozygotes, pointing out the essential role of *Sar1b* in mouse embryonic development. In a second step, we demonstrated that disrupting only one *Sar1b* allele causes significant lipid intestinal malabsorption because of CM secretion failure, which establishes the cause-effect association of *Sar1b* defects and CRD. Subsequently, viable heterozygotes exhibit a decrease in the intestinal protein expression of Apo B and MTTP, presumably contributing to the decrease of CM output. Further experiments showed perturbations of lipid homeostasis (e.g., high FA β-oxidation and low lipogenesis) as a consequence of *Sar1b* abnormalities. Besides, defective *Sar1b* lowers HDL-C biogenesis by affecting mRNA expression of Apo A-I and ABCA1 in the intestine. Another highlight of the present study includes the little effect of *Sar1b* alterations on the liver (compared with the intestine).

During our analysis of the genotype of 21-day-old mice from heterozygote crosses, we noted a total absence of homozygous mice, which is not consistent with the 1:2:1 Mendelian frequency ratios of WT:heterozygotes:homozygotes. The reason for the deficiency of homozygotes is certainly related to embryonic lethality as was the case for homozygous mouse models of Apo B and MTTP mutations (31, 32). Such studies have pointed to the possibility of deficient yolk sac lipoprotein production, which may reduce the delivery of lipid nutrients to the developing embryo at a critical developmental period. Still others cited neurodevelopmental abnormalities, which are characterized by exencephalus and hydrocephalus (32, 33) or defects in erythropoiesis, known to lead to mid-gestational embryonic death (34). But regardless of the etiopathogenesis, homozygous mouse models of hypobetalipoproteinemia, abetalipoproteinemia, and CRD share embryonic lethality, which emphasizes the critical importance of their respective proteins Apo B, MTTP, and Sar1b. Whether homozygous *Sar1b*^*-/-*^ mice are not viable because of a defect in embryonic brain development deserves complementary studies. It has already been known that Sar1b is abundant in the brain and its deletion in zebrafish embryos cause absence of neuroD-positive neurons (21). On the other hand, *Sar1b* knockdown in the developing cerebral cortex impedes the radial migration and axon elongation of the cortical neurons in mice (22). It remains important to determine the contribution not only of intestinal lipid malabsorption to neuronal disorders, and vice versa, but also the influence of each on embryogenesis processes. Efforts are also required to understand the reasons why mice fail to give birth to homozygotes with defective Sar1b when humans have this ability. Definitely, further research is warranted to investigate the gestational age and mechanisms of embryonic lethality in our two *Sar1b*^*mut/+*^ and *Sar1b*^*del/+*^ mice.

The difference in lethality between mice and humans is intriguing. This divergence has already been observed in other congenital intestinal fat malabsorption such as hypobetalipoproteinemia and abetalipoproteinemia (31, 35). Furthermore, the species heterogeneity was as well noted in a plethora of additional diseases (36, 37, 38). Although the causes are still poorly understood, plausible explanations may be proposed. First of all, it has been reported that human and mice exhibit considerable differences in development and morphology (e.g., gastrulation and neurulation) (39, 40). Even global expression profiling in physiological and pathophysiological conditions indicated changes in genetic programs behind significant human-mouse differences in embryogenesis (39).

Another meaningful point is the important differences in the distinctive role of the yolk sac between mice and human. In mice, the secretion of Apo B-containing lipoproteins from the yolk sac is essential for the survival of the embryo (41), since the deletion of *ApoB* and *MTTP* causes embryo death and high accumulation of lipids in the yolk sac (31, 35), which is not the case for humans. Even though the placenta can secrete Apo B lipoproteins and the yolk sac does express Apo B (42, 43), the absence of lipoprotein formation is not lethal in humans since TGs are available to the fetus from other sources (44). In fact, the yolk sac in humans, compared with mice, is a rudimentary organ that rapidly loses its nutritional functions in the early embryogenesis, but FAs bound to albumin can cross the placental barrier by diffusion (45). Another possible difference between mice and humans refers to the significant role of *Sar1b* in the brain of mice (22). Finally, it is possible that Sar1b activity is pivotal given the limited compensation by its *Sar1a* isoform in mice, contrary to human (19). Accordingly, Sar1a gene expression increases in the intestine of patients with CRD (15).

RT-qPCR and Western blot were used to confirm heterozygosity by evaluating Sar1b mRNA and protein mass in intestinal and hepatic tissue samples. As expected, Sar1b gene and protein expressions in *Sar1b*^del/+^ mice showed about half the value of control WT mice. However, even if we anticipated no changes in Sar1b gene and protein expressions in the framework of testing mice with the point mutation, we surprisingly noticed that the protein expression was reduced by half. Our assumption is that the point mutation promoted Sar1b protein instability and proteasomal degradation. It is possible that the point mutation led to inappropriate Sar1b protein folding, which was monitored through the ER quality control mechanisms, leading to ER-associated protein degradation (46, 47). It is noteworthy that misfolded proteins are eliminated after being recognized, retrotranslocated into the cytosol, polyubiquitinated, and then extracted from the ER membrane to be degraded in the cytosol by the ubiquitin/proteasomal system (48). In particular, genetic mutations of secretory proteins result in their accumulation in the ER lumen followed by premature ER-associated protein degradation, a strategy largely implicated in various pathologies such as hereditary hemorrhagic telangiectasia type 2 (49, 50). Such observations have been reported in tumoral calcinosis where the missense mutation of KL1 gene led to a change from histidine to arginine, thereby destabilizing the glycosides catalytic domain while lessening KL1 protein production (51). A similar metabolic protein fate was also observed in PTEN mutants and Miller syndrome (52).

We decided to use 12-week-old mice because we wanted to avoid experiments with old mice since CRD begins in the earliest months and years of life. In fact, there were only 4 adults among the 62 very young patients reported in the literature (13, 53). These adults must certainly have escaped an early and precise diagnosis despite their symptoms. Of importance, the characteristic CRD phenotypes were already apparent in 12-week-old mice (25). It is also important to note that many studies on congenital fat malabsorption diseases (e.g., hypobetalipoproteinemia and abetalipoproteinemia) used animal mouse models of the same age (32, 42, 54, 55, 56).

Packaging of intestinal CM and hepatic VLDL has been the subject of intense research over the last four decades. Their assembly has in common several key proteins and critical processes. First, Apo B provides the structural framework for their formation, but CM contains Apo B-48, representing 48% of the Apo B-100 within hepatic VLDL. Second, the resident endoluminal ER protein MTTP physically transfers the lipid components to the nascent Apo B to initiate the assembly of CM and VLDL, which then undergo progressive modification and vesicular transport to the Golgi apparatus. Third, the translocation of CM and VLDL to the Golgi requires Sar1b protein to initiate vesicle formation for COPII-dependent transport to the Golgi. Fourth, mutations in *Apo B*, *MTTP*, and *Sar1b* completely interrupt initiation stages of pre-CM and VLDL formation in the case of the two former genes and blocks the maturation and exit of CM from the ER toward the Golgi in the case of the last gene. Therefore, congenital defects lead to intestinal fat malabsorption and VLDL delivery failure. However, unexpectedly, only sporadic and very limited steatosis was noticed in few patients with CRD despite *Sar1b* aberrations (2, 18, 57). Moreover, contrary to the total absence of small intestine-derived CM in CRD in response to a fat meal test, the level of VLDL of hepatic origin was similar to that of control human participants. In line with these observations, our two *Sar1b*^*mut/+*^ and *Sar1b*^*del/+*^ mouse models display a similar behavior in view of little liver damage. Indeed, no significant hepatic lipid accumulation and abnormal lipid metabolism were observed despite genetically modified *Sar1b*. Nevertheless, the little impact of Sar1b mutations on VLDL delivery by the liver of patients with CRD and mice compared with McArdle-RH7777 hepatocytes (20) is an intriguing issue, which needs clarifications in future investigations.

As mentioned above, our *Sar1b*^*mut/+*^ and *Sar1b*^*del/+*^ mouse models recapitulated intestinal fat malabsorption and CM secretion defects, which are the most dominant features of patients with CRD. Probably, these mutations disable the formation of COPII and thus block the transport of CM from the ER to the Golgi complex, resulting in an increased deposition of lipids in the mucosal cells of the proximal small intestine and, hence, decreased plasma lipids concentration. Of interest, *Sar1b*^*mut/+*^ and *Sar1b*^*del/+*^ mice displayed intestinal depletion of MTTP and Apo B protein expression, which may contribute to fat malabsorption. This is consistent with the decreased Apo B protein expression in response to *Sar1b* gene silencing in Caco-2/15 cells recently reported, using the zinc finger nuclease technique (19). It is necessary to investigate whether the accumulation of defective Sar1b protein induces ER stress, which triggers the ubiquitin-proteasome system with a potency to degrade Apo B (58, 59).

The finding of HDL-C decline, in our genetically *Sar1b-*modified mice, led us to examine Apo A-I and ABCA1, two critical proteins for early HDL biogenesis. As well established, newly secreted Apo A-I is lipidated by phospholipids and CHOL, primarily stimulated by membrane protein ABCA1 at the cell surface (60, 61). In our experiments, the low gene expression of Apo A-I and ABCA1 in the gut resulted in low levels of HDL-C in animals with CRD. These results not only confirm the data previously obtained in intestinal Caco-2/15 cells in response to *Sar1b* deletion (2, 23, 57) but might also explain the severely reduced CHOL content of the HDL fraction of patients with CRD (7, 57). A reduction in Apo A-1 and HDL secretion was also observed in patients with a deletion of Apo B (62) and MTTP (63), but the mechanisms are still unknown. Whether increased catabolism of Apo A-I and HDL constitutes an additional mechanism for the low HDL-C levels might be an avenue to explore further.

In conclusion, we have established two novel animal models that recapitulate various features of congenital CRD, including CM secretion failure, hypocholesterolemia, and hypoalphalipoproteinemia (low HDL levels). In addition, among the mouse model disturbances that could not be scrutinized in humans given the limitation of tissues, we have noticed perturbations in lipid homeostasis, such as FA β-oxidation and lipogenesis, due to abnormal status of powerful transcription factors. In addition, the liver did not display the same metabolic behavior as the intestine despite the defect in its intrinsic *Sar1b* gene. Another central finding of our study is related to embryonic lethality of homozygous mice with *Sar1b* genetic defects as the breeding of heterozygous mice yielded no viable homozygotes, which indicates the essential role of the Sar1b protein in mouse embryonic development.

## Data availability

All data supporting the findings of this study are contained in the article and in the supplemental data.

## Supplemental data

This article contains supplemental data.

## Conflict of interest

The authors declare that they have no conflicts of interest with the contents of this article.
