# Supplementary material for: Sar1b mutant mice recapitulate gastrointestinal abnormalities associated with chylomicron retention disease
Source: J Lipid Res. 2021 May 5;62:100085. doi: 10.1016/j.jlr.2021.100085 (PMC8175419; doi:10.1016/j.jlr.2021.100085)
Supplement: Supplemental data [file mmc1.pdf]

SAR1B MUTANT MICE RECAPITULATE GASTROINTESTINAL ABNORMALITIES ASSOCIATED WITH CHYLOMICRON RETENTION DISEASE

Nickolas Auclair[1,2] Alain T. Sané[1], Lena Ahmarani[1,3], Nathalie Patey[1,4], Jean-François Beaulieu[5], Noel Peretti[6] Schohraya spahis[1,3], Emile Levy[1,2,3*]

[1]Research Center, CHU Ste-Justine, Departments of [2]Pharmacology & Physiology, [3]Nutrition and [4]Pathology, Université de Montréal, Montreal, Quebec, Canada, H3T 1C5 [4]Laboratory of Intestinal Physiopathology, Department of Immunology and Cell Biology, Faculty of Medicine and Health Sciences, Université de Sherbrooke, Sherbrooke, QC J1H 5N4, Canada. [6]Department of Pediatric Gastroenterology-Hepatology and Nutrition, Laboratory INSERM 1060 Cardiovascular Metabolism Endocrinology and Nutrition CarMEN, Lyon, France

Address for correspondence: *Dr. Emile Levy
GI-Nutrition Unit
CHU Sainte-Justine
3175 Ste-Catherine Road #4.17.005
Montreal, Quebec, Canada, H3T 1C5
Tel.: (514) 345-7783
E-mail: emile.levy.hsj@ssss.gouv.qc.ca

SUPPLEMENTARY METHODS

Generation of Sar1b KO *(Sar1b$^{del/+}$)* and mutated *(Sar1b$^{mut/+}$)* mice

Embryos were produced from C57BL/6N mice (Charles River Laboratories), and CD-1 female mice served for embryo transfer. The CD-1 line is robust and largely used for genetic experiments because of their efficient breeding and large litter sizes. All animal care and procedures performed in this study were approved by the Research Center platform of the Centre Hospitalier de l'Université de Montréal (CRCHUM) animal care committee in accordance with the guidelines from the Canadian Council on Animal Care in science (CCAC).

Preparation of CRISPR-Cas9 electroporation mixes (*Sar1b*$^{del/+}$)

The Cas9 protein (Integrated DNA technologies (IDT, # 1081058), custom crRNA (IDT, Alt-R^{TM}crRNA) and generic tracrRNA (IDT # 1072533) were prepared as previously described.(16) Briefly, CRISPR RNA-Trans-activating CRISPR RNA complex paired-guide RNA (pgRNA) was formed by mixing an equimolar ratio of the aforementioned components, which were incubated 5 minutes at 95°C and allowed to cool down to room temperature (10 minutes). The Cas9 Ribonucleoprotein (Cas9 RNP) complex was assembled by incubating 20 µmoles of Cas9 protein with 20 µmoles of assembled pgRNA for 10 minutes at room temperature. The RNP complexes were then combined in 20 µl Opti-MEM at a final concentration of 4 µM (ThermoFisher Scientific #31985070).

Preparation of CRISPR-Cas9 electroporation mixes (*Sar1b*$^{mut/+}$)

The Cas9 RNP complex was assembled as previously described.(16) Briefly, Cas9 protein (80 µmoles) was incubated with assembled pgRNA (80 µmoles) and incubated for 10 minutes at room temperature. The RNP complex was then combined in 20 µl Opti-MEM at a final concentration of 4 µM (Thermo Fisher Scientific # 31985070) along with 10 µM of repair template (custom Ultramer ssDNA, IDT).

Zygote preparation

Prepubescent 3 weeks old C57BL/6 females were superovulated 67 hours prior zygote collection by 5 IU intraperitoneal administration of pregnant mare serum gonadotrophin (Genway Biotech Inc, GWB-2AE30A) followed 47-48 hours later with 5 IU of human chorionic gonadotrophin (Sigma-Aldrich, CG10-1VL) before being bred. Fertilized 1-cell stage embryos were collected and kept in embryomax KSOM advance media (Millipore Sigma) at 37°C under 5% CO_2 until electroporation.

Electroporation procedure

Briefly, 1-cell embryos were washed in batch of 50 through 5 drops of M2 media before being rinsed in a single drop of Opti-MEM. The embryos were then transferred to the 20 ul electroporation mix. The solution was then transposed to a pre-warmed 1-mm cuvette (BioRad). Electroporation was carried out using a Gene Pulser XCell electroporator with the following conditions: 30V, 3 ms pulse duration, 2 pulse 100 ms interval. Electroporated embryos were recovered from the cuvette and washed in three drops of embryomax KSOM advance media before being incubated a least one hour at 37°C under 5% CO_2 prior implantation in pseudopregnant females. Surviving embryos, up to the 2-cell stage, were implanted in pseudo pregnant females (0.5 dpc).

Genotyping

Briefly, ear biopsies from 21-day old mice were digested in MyTaq Extract PCR kit according to the manufacturer (Bioline, # BIO-21126). PCR amplification was performed using the high-fidelity enzyme Q5 from New England Biolab (# M0530L) with locus specific primers (*Sar1b*$^{del/+}$): [(CATCACACATGGCTCTTCATT and TGATGTGCTCAGTTCAGTCCTT, and

ACTTCGGCTTTGCTCTTTGTAG and AAAGGCTTCACCTCTCCATACA)]; (*Sar1b*$^{mut/+}$): [(GACTGAGTCCTTGGCTATTTGG and CCTTTCTTAAGCTGGGTATGGA)]. PCR products were gel-electrophoresed to assess for zygosity through visualization of specific bands and the desired mutation presence was confirmed by sequence alignment using the Snapgene software.

List of all primers used for RT-qPCR analysis

	**Primers**
**ApoB**	Forward: 5-TGAATGCACGGGCAATGA-3
	Reverse: 5-GGCATTACTTGTTCCATGGTTCT-3
**MTTP**	Forward: 5-ATGATCCTCTTGGCAGTGCTT-3
	Reverse: 5-TGAGAGGCCAGTTGTGTGAC-3
**ApoA1**	Forward: 5-CCACACCCTTCAGGATGAAAG-3
	Reverse: 5-TGGCTCCCTGTCAGGAAGAC-3
**ABCA1**	Forward: 5-AGGGTTTCTTTGCTCAGATTGTC-3
	Reverse: 5-TGCCAAAGGGTGGCACA-3
**SREBP1**	Forward: 5-GACCCTACGAAGTGCACACA-3
	Reverse: 5-TCATGCCCTCCATAGACACA-3
**ACC**	Forward: 5-TGGCTTCTCCAGCAGAATTT-3
	Reverse: 5-AGATCGCATGCATTTCACTG-3

SUPPLEMENTARY FIGURE LEGEND

FIGURE S1 **Impact of *Sar1b* deletion and mutation on body weight, food intake and weight of several organs.** After weaning, WT (Ctrl), heterozygous *Sar1b*$^{mut/+}$ and *Sar1b*$^{del/+}$ mice were fed ad libitum with a conventional chow diet. **(A)** Body weight and **(B)** food intake was recorded every day for 1 week. Prior to the sacrifice, mice were fasted for 6 hours followed by an oral gavage of olive oil (200 μL). **(C)** Total adipose tissue, **(D)** stomach, **(E-G)** small intestine, **(H-I)** colon, **(J)** liver, **(K)** heart and **(L)** brain tissues were weighted. Results represent the means ± SEM of 10-13 mice (9-11 weeks old) in each group.

Figure S1

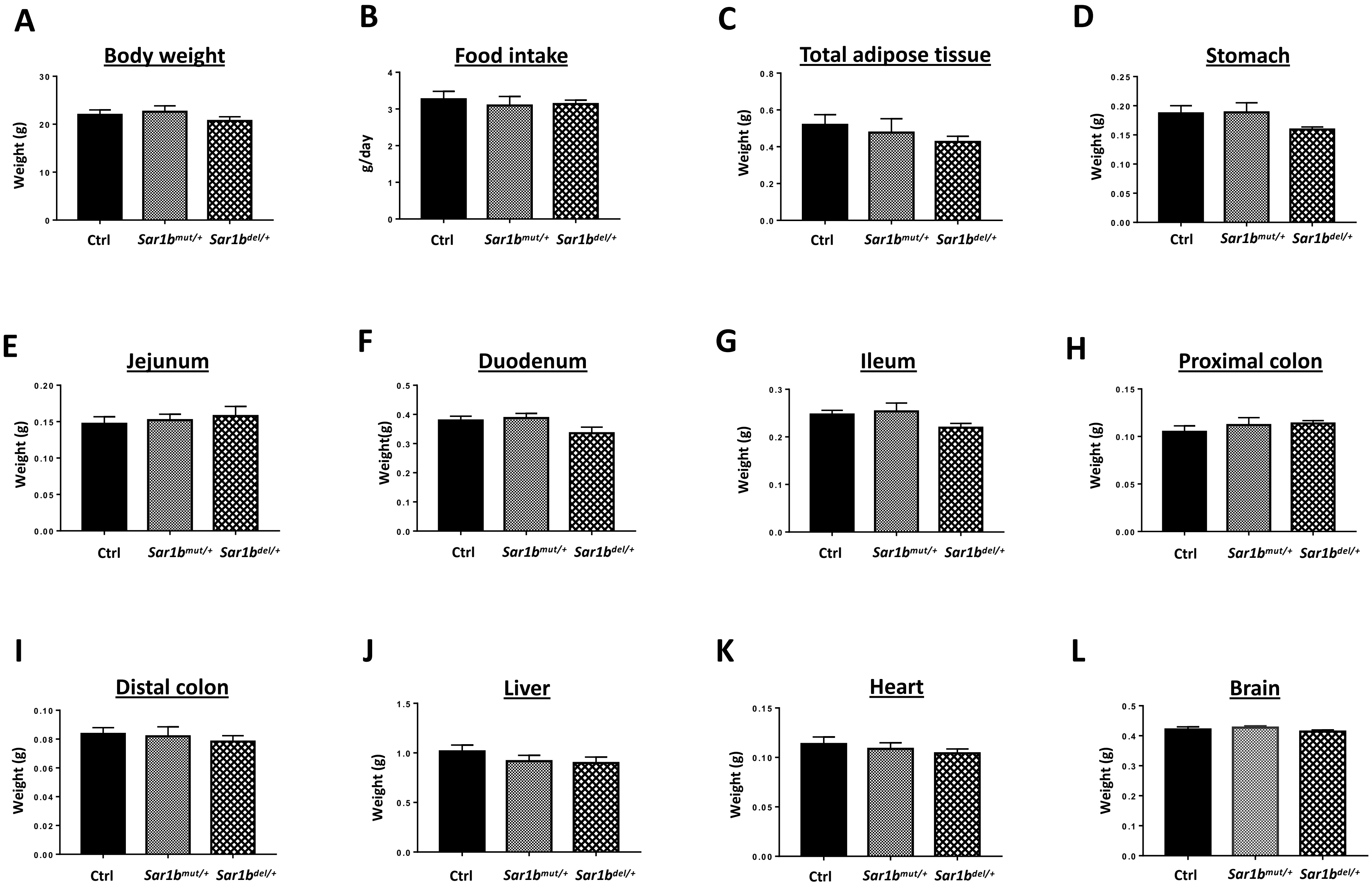
A
Body weight
Weight (g)
0
10
20
30
Ctrl
Sar1b$^{mut/+}$
Sar1b$^{del/+}$
B
Food intake
g/day
0
1
2
3
4
Ctrl
Sar1b$^{mut/+}$
Sar1b$^{del/+}$
C
Total adipose tissue
Weight (g)
0.0
0.2
0.4
0.6
0.8
Ctrl
Sar1b$^{mut/+}$
Sar1b$^{del/+}$
D
Stomach
Weight (g)
0.00
0.05
0.10
0.15
0.20
0.25
Ctrl
Sar1b$^{mut/+}$
Sar1b$^{del/+}$
E
Jejunum
Weight (g)
0.00
0.05
0.10
0.15
0.20
Ctrl
Sar1b$^{mut/+}$
Sar1b$^{del/+}$
F
Duodenum
Weight(g)
0.0
0.1
0.2
0.3
0.4
0.5
Ctrl
Sar1b$^{mut/+}$
Sar1b$^{del/+}$
G
Ileum
Weight (g)
0.0
0.1
0.2
0.3
Ctrl
Sar1b$^{mut/+}$
Sar1b$^{del/+}$
H
Proximal colon
Weight (g)
0.00
0.05
0.10
0.15
Ctrl
Sar1b$^{mut/+}$
Sar1b$^{del/+}$
I
Distal colon
Weight (g)
0.00
0.02
0.04
0.06
0.08
0.10
Ctrl
Sar1b$^{mut/+}$
Sar1b$^{del/+}$
J
Liver
Weight (g)
0.0
0.5
1.0
1.5
Ctrl
Sar1b$^{mut/+}$
Sar1b$^{del/+}$
K
Heart
Weight (g)
0.00
0.05
0.10
0.15
Ctrl
Sar1b$^{mut/+}$
Sar1b$^{del/+}$
L
Brain
Weight (g)
0.0
0.1
0.2
0.3
0.4
0.5
Ctrl
Sar1b$^{mut/+}$
Sar1b$^{del/+}$
